# Laser Hair Removal With Alexandrite Laser: Tailoring Brushing and Stamping Protocols for Optimal Comfort and Efficiency

**DOI:** 10.1111/jocd.71150

**Published:** 2026-08-30

**Authors:** Anže Zorman

**Affiliations:** ^1^ Medilase d.o.o Ljubljana Slovenia; ^2^ Fotona d.o.o Ljubljana Slovenia

## Abstract

**Objectives:**

The study aimed to evaluate the safety and efficacy of the Avalanche brushing protocol across various body regions, assess the clinical outcomes of the combined brushing‐stamping treatment scheme, and compare patient‐reported comfort levels between the two approaches.

**Methods:**

Sixty‐one distinct paired treatment areas were treated. The left side received the brushing protocol exclusively across all sessions, whereas the right side underwent a combined regimen: brushing for the initial three sessions, followed by stamping for the remaining three. Alexandrite (755 nm) laser was used.

**Results:**

The mean hair reduction score was 3.02 (Avalanche method) and 2.98 (stamping method) at the 6‐month follow‐up. Both treatment methods offered low discomfort; the brushing technique (2.2/10) was associated with significantly lower (*p* < 0.05) patient discomfort compared to stamping (2.7/10).

**Conclusions:**

We recommend initiating hair removal with the brushing protocol in patients exhibiting increased sensitivity and/or thick, dense hair, particularly during the early treatment sessions. As hair density and thickness diminish over the course of therapy, transitioning to the stamping protocol may be appropriate.

## 1 Introduction

Unwanted hair is a common aesthetic concern across many cultures [1]. Although excessive hair growth may be entirely natural, it can also result from medication side effects or signal an underlying medical condition [2, 3]. Achieving a smooth, hair‐free appearance remains a challenge, with significant effort and resources dedicated to various hair removal methods. Traditional treatments such as shaving, plucking, waxing, chemical depilatories, and electrolysis can be considered tedious and painful, and most only produce short‐term results.

Laser and intense pulsed light (IPL) based hair removal have become one of the most widely used phototherapy methods [4, 5]. Among these, laser therapy stands out as the safest and most effective option for achieving long‐term hair removal. By targeting hair follicles with thermal energy, lasers achieve precise destruction while minimizing damage to surrounding tissues—a principle known as selective photothermolysis [6, 7]. Different wavelengths have been used for hair removal [1]. Long‐pulse ruby (694 nm), long‐pulse alexandrite (755 nm), long‐pulse diode (810 nm), long‐pulse Nd:YAG (1064 nm), and intense pulsed light (IPL) (590–1200 nm) destroy hair photothermally through melanin absorption. Some researchers have also investigated techniques that combine multiple wavelengths simultaneously to enhance efficacy [8].

In recent years, various laser hair removal techniques have been tested in pursuit of more effective yet comfortable treatment options. The traditional approach, characterized by high fluence, low repetition rate, and a single‐pass method (so called “stamping”), has been compared to the brushing/painting mode, which utilizes high frequency, low fluence, and a multiple‐pass technique [9]. Studies have shown that both methods yield equally effective results, while the latter offers a more comfortable experience for patients [10, 11, 12, 13, 14]. These, however, may prolong the treatment time because several passes are needed for effective hair removal.

Traditionally, laser hair removal devices were limited to supporting either the stamping or brushing protocol. Recent advancements in device technology have introduced systems capable of performing both, allowing clinicians to tailor treatments according to the anatomical area and individual patient sensitivity.

This study was designed to compare two treatment strategies applied simultaneously on opposite sides of the body. The brushing protocol, referred to as the Avalanche approach, was used exclusively on the left side throughout all six hair removal sessions. On the right side, a combined treatment scheme was employed: the brushing protocol was used for the first three sessions—targeting the phase when hair density and thickness were highest and patients most frequently reported discomfort—followed by three sessions using the stamping protocol, which is generally faster and better tolerated in later stages of treatment. This split‐body design allowed for direct intra‐individual comparison, made possible by the versatility of next‐generation high‐power alexandrite laser systems.

The study aimed to evaluate the safety and efficacy of the Avalanche brushing protocol across various body regions, assess the clinical outcomes of the combined brushing‐stamping treatment scheme, and compare patient‐reported comfort levels between the two approaches.

## 2 Material and Methods

### 2.1 Laser System

The AvalancheLase LXP laser system (Fotona, d.o.o., Ljubljana) utilized in this study integrates dual laser sources: alexandrite (755 nm) and neodymium‐doped yttrium aluminium garnet (Nd:YAG, 1064 nm). However, only the alexandrite wavelength (755 nm) was employed for experimental procedures. The device incorporates integrated cooling technology, which operates through an atomized pulsed liquid spray system governed by a digitally controlled mixture of liquid (water) and gas (air). This mechanism facilitates dry molecular cooling (*DMC*), wherein rapid evaporation of molecular droplets ensures efficient thermal regulation, thereby mitigating epidermal heating effects [12, 15].

### 2.2 Study Population and Clinical Protocol

Thirty‐one patients asking for hair removal between May 2023 and August 2024 at Medilase Laser Center, Ljubljana, Slovenia were included in the study. All patients signed informed consent before enrolment. Most patients consented to photographic documentation throughout the treatment; however, a minority declined photography of intimate areas. The inclusion criteria were: age above 18 years old, willingness to complete the treatment sessions and follow‐ups, non‐waxed or non‐shaved treatment area 6 weeks prior to the first treatment. Exclusion criteria were as follows: pregnancy, previous laser hair removal in the treatment area, and PCOS. The research followed the principles outlined in the Declaration of Helsinki.

Patients were between 20 and 55 years old with skin Types II–IV; 16 patients FP II, 9 patients FP III, and 6 patients FP IV. At the initial consultation, 23 patients presented with thin hair, whereas 8 had thick hair. The majority had black hair (20/31), followed by brown or orange hair (7/31), and a smaller proportion had light‐colored hair. The study participants were instructed to shave the hair at the treatment area 2–3 days before the scheduled procedure or it was done right before the start of the laser session. Each patient received a total of six treatment sessions at 1 month interval (bikini, armpits) or 2 months interval (upper leg, lower leg, buttocks). The left side was treated with the Avalanche/brushing protocol in all 6 sessions while the right treatment side was treated with the Avalanche/brushing protocol in sessions 1–3 and with the stamping protocol in sessions 4–6. A detailed description of the treatments' parameters is shown in Table 1.

**TABLE 1:** Average parameters used in each session.

Left side/Avalanche approach					Right side/Combined approach				
Protocol	Spot size (mm)^a^	Fluence (J/cm^2^)	Frequency (Hz)^b^	Pulse duration (ms)	Protocol	Spot size (mm)^a^	Fluence (J/cm^2^)	Frequency (Hz)	Pulse duration (ms)
1. Avalanche	12/20	8–9	5–7	2–3	Avalanche	12/20	8–9	5–7	2–3
2. Avalanche	12/20	9–10	3–7	2–3	Avalanche	12/20	9–10	3–7	2–3
3. Avalanche	12/20	10–11	3–7	2–3	Avalanche	12/20	10–11	3–7	2–3
4. Avalanche	12/20	11–12	3–7	2–3	Stamping	12/20	15–17	2–3	2–3
5. Avalanche	12/20	12–13	3–7	2–3	Stamping	12/20	13–18	2–3	2–3
6. Avalanche	12/20	13–14	3–7	2–3	Stamping	12/20	14–18	2–3	2–3

^a^ 12 mm spot size for bikini and armpits, 20 mm spot size for other areas.

^b^ 3 Hz with 20 mm spot size, 6–7 Hz with 12 mm spot size.

A preset cumulative energy per treatment area was applied when using the Avalanche brushing protocol according to the manufacturer recommendations. In contrast, the stamping protocol allowed for variable energy output. Table 2 presents the preset energy values used in Avalanche mode, alongside the average cumulative energy delivered during stamping sessions for each body region.

**TABLE 2:** Cumulative energy of utilized treatment protocols.

Per (one) side	Avalanche mode	Stamping mode
	Preset cumulative energy (*J*)	Average delivered cumulative energy (*J*)
Bikini	7000	3500
Armpits	3750	2600
Upper leg	45 000	25–30 000
Lower leg	40 000	30–35 000

The initial fluence settings were based primarily on the manufacturer's recommendations and supplemented by the operator's clinical experience, taking into account each patient's Fitzpatrick skin type and individual sensitivity.

All treatment sessions incorporated the DMC cooling technique, utilizing spray settings of water at levels 4–5 and air at level 5. High‐resolution digital photographs of the treated areas were obtained at baseline, as well as at 3‐month and 6‐month follow‐up intervals after the last session.

### 2.3 Outcome Measures

Pain was assessed during each treatment by a 0–10 VAS scale, 0 indicating no pain and 10 indicating the worst imaginable pain.

Photographic evaluations were conducted for all patients who completed the 6‐month follow–up and consented to image documentation. The assessments were performed independently by three blinded evaluators representing diverse perspectives: a licensed medical doctor, a layperson, and a certified beautician using the scoring system shown in Table 3.

**TABLE 3:** Four‐point hair reduction scale used for assessment.

	1	2	3	4
Hair reduction	0%–25%	26%–50%	51%–75%	76%–100%

In addition to clinical evaluations, patient‐reported outcomes were assessed to gauge subjective treatment satisfaction. Each participant rated their personal satisfaction using the 7‐point Patient's Global Impression of Improvement (PGI‐I) scale at 3‐ and 6‐months follow–up after last session. Furthermore, patients were also asked to indicate their preferred treatment method between the two available techniques: Avalanche and stamping. Statistical analysis was performed using Prism version 10.2.2 (GraphPad, California, USA). A repeated measures one‐way ANOVA followed by Šidák's post hoc test was applied to assess statistical significance.

## 3 Results

All 31 enrolled patients successfully completed both the 3‐month and 6‐month follow‐up assessments, covering a total of 61 distinct paired treatment areas. The most frequently treated regions included the bikini area (*n* = 23; 38%), followed by the axillae (*n* = 19; 31%), lower legs (*n* = 13; 21%), upper legs (*n* = 5; 8%), and buttocks (*n* = 1; 2%). Due to constraints in photographic consent—particularly concerning intimate areas—50 paired sets of baseline and follow‐up images were available for evaluative analysis.

At the 6‐month follow‐up, the mean hair reduction score, based on the scoring criteria outlined in Table 3, was 3.02 for areas treated with the Avalanche method and 2.98 for sites where stamping was used during the final three sessions, when considering all areas combined. Table 4 presents the per‐region hair‐reduction scores. Per‐region statistical analysis was not performed for the upper legs (*n* = 5) and buttocks (*n* = 1) due to the small number of treated sites. There were no statistically significant differences observed between the left and right treatment sides, details in Table 4.

**TABLE 4:** Average improvement per side for each region at the 6‐month follow‐up, as measured by hair reduction score.

	Bikini		Armpits		Lower legs	
	Avalanche	Stamping	Avalanche	Stamping	Avalanche	Stamping
Hair reduction	2.73	2.78	2.82	2.71	3.50	3.45

**FIGURE 1:**
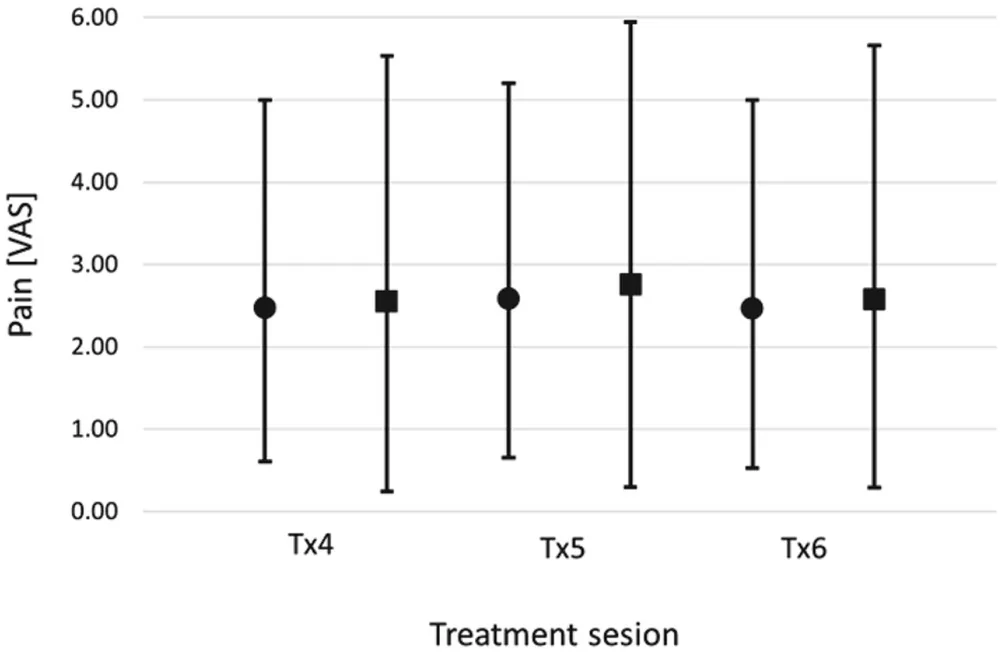
Plot showing the distribution of VAS Pain Scores in the 4th, 5th, and 6th session of hair removal when both methods of laser application (Avalanche and Stamping) were employed. Plot denotes mean value with 95% CI (dots represent Avalanche and squares Stamping).

Patients' assessments of treatment‐associated pain are illustrated in Figure 1. Aggregate data revealed that the Avalanche technique was consistently associated with significantly lower pain scores (*p* = 0.0006) compared to stamping across all sessions. The mean effect between the groups was 0.48 (95% [0.21, 0.76]).

Stratified analysis demonstrated the most pronounced differences in the bikini area (*p* < 0.01) and armpits (*p* < 0.05), while no statistically significant differences were noted for the lower legs and thighs. Importantly, pain scores did not differ between left and right side during the first three sessions, during which the Avalanche protocol was uniformly applied.

Patient‐reported outcomes reflected high levels of satisfaction, independent of the treatment modality employed (Avalanche vs. Stamping). PGI‐I scores remained consistent at both follow‐up intervals: 1.75 (± 0.84) at 3‐ and 6‐months follow‐up.

A majority of participants (87%) expressed no preference between the two treatment modalities. Two patients (6.5%) favored the stamping technique, whereas two others (6.5%) preferred the Avalanche protocol. No serious adverse effects were reported throughout the study period. Approximately 20% of participants experienced expected immediate responses—transient erythema and perifollicular oedema, both of which resolved spontaneously within 24 h or less. No evidence of burns, hypo‐ or hyperpigmentation, blistering, crusting, scarring, ecchymosis, purpura, folliculitis, or other skin infections was observed. Additionally, adverse effects reported in some hair removal studies—including dry skin, allergic or chemical reactions, paradoxical hypertrichosis, accelerated skin aging, tissue injury, and bleeding—were also not noted in this case.

## 4 Discussion

The efficacy of long‐pulsed 755 nm alexandrite laser hair removal was first reported by Finkel et al. [16]. Although hair removal using 755 nm alexandrite and other wavelengths is well‐established, it is frequently accompanied by notable pain and discomfort. This study aimed to evaluate two distinct protocols for integrating the Avalanche brushing technique into a laser‐based hair removal regimen. The first protocol employed the brushing technique across all six treatment sessions. The second protocol applied brushing during the initial three sessions—targeting the early phase when pain sensitivity is typically highest—and transitioned to stamping for the final three sessions, which are generally faster and better tolerated in later stages. This hybrid approach was facilitated by the capabilities of novel high‐power alexandrite laser systems. The objective was to assess whether this sequential treatment strategy could enhance patient comfort while optimizing overall treatment duration.

**FIGURE 2:**
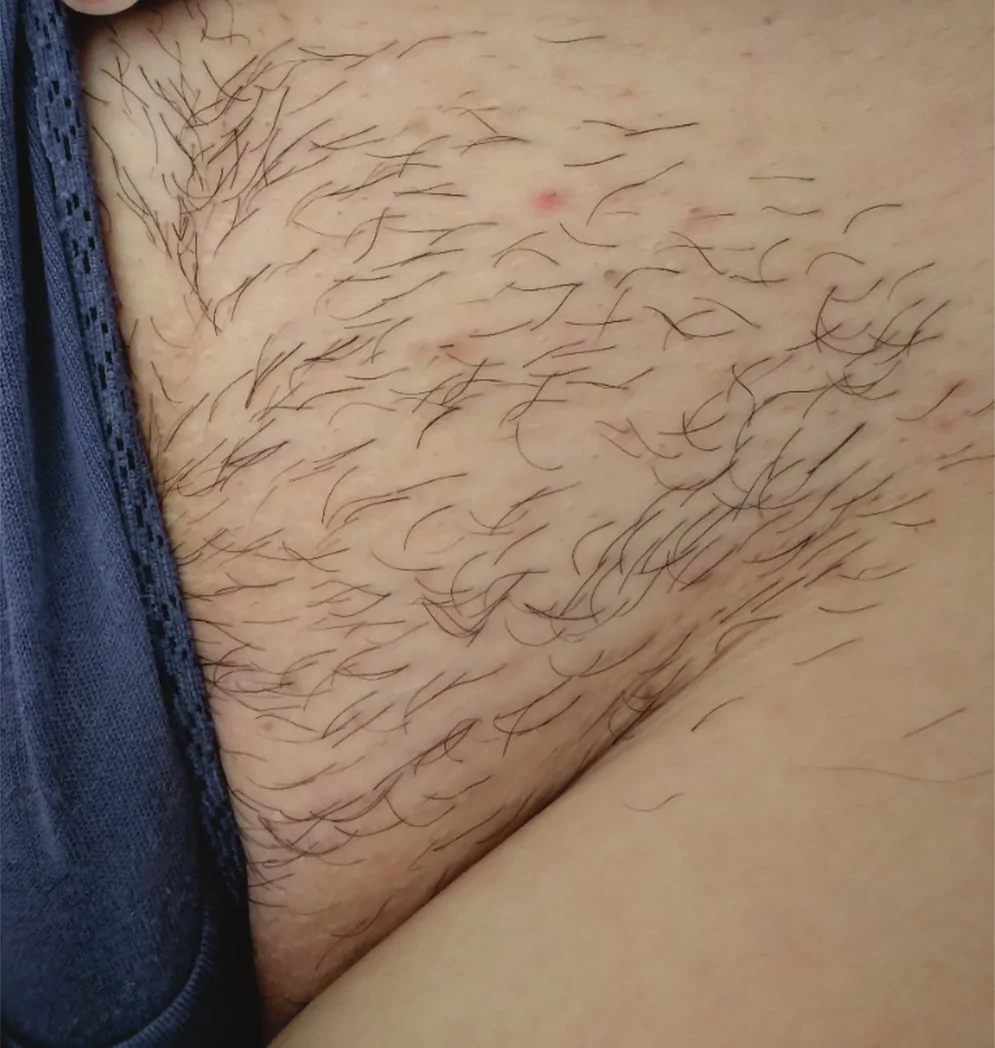
Left side of bikini area in patient X before the 1st treatment.

**FIGURE 3:**
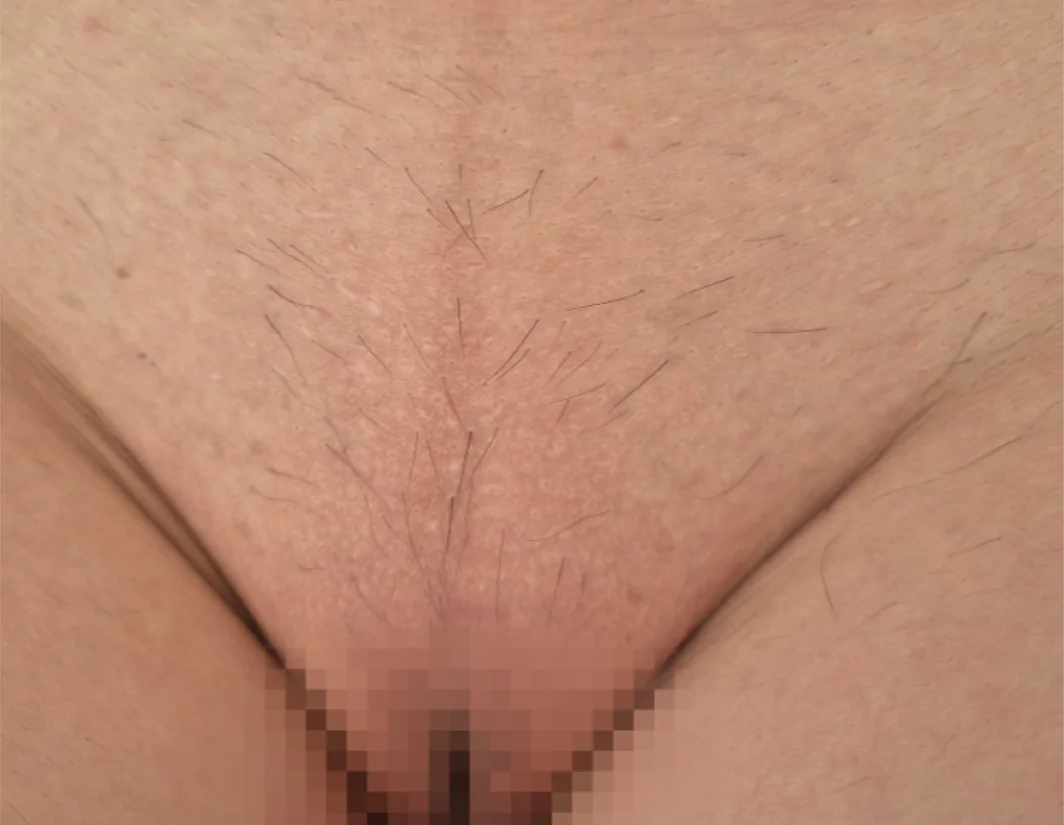
Both sides of bikini area in patient X 6 months after 6 treatment sessions.

The study has shown that Avalanche (brushing) mode has proven to be a safe and effective technique, yielding outcomes comparable to the traditional stamping method, see Figures 2 and 3. This finding is further supported by the low mean pain scores recorded on the Visual Analog Scale (VAS), with Avalanche mode averaging 2.2 and stamping mode 2.7. The difference between these modalities was statistically significant, highlighting the superior tolerability of Avalanche mode. The pain levels were low overall in both treatment groups, which could be attributed to the innovative DMC cooling system which has been shown to provide efficient cooling with a prolonged effect compared to other cooling methods [15].

The fluence used in stamping mode on the right side (sessions 4–6) falls toward the lower end of the published fluence spectrum [1], and this was intentional. In our earlier, real‐world experience with the same device (unpublished), higher fluences consistently produced unwanted side effects. To minimize these risks, we deliberately selected more conservative parameters. Although higher fluence might have produced even greater improvement—particularly in comparison with the left side—it would almost certainly have resulted in higher VAS scores, which we aimed to avoid.

Moftah et al. [14] is the only study reporting lower VAS pain scores, which can be attributed to the use of the brushing technique combined with substantially lower fluence compared with our protocol. The absolute VAS values observed in our study are partly influenced by the fact that all areas underwent three initial brushing sessions, which likely reduced hair density and thickness before sessions 4–6. Consequently, the recorded VAS scores primarily reflect patient comfort during these later sessions, when hair burden was already diminished.

In contrast, other comparative studies consistently documented higher levels of patient discomfort. For example, Piccolo [17] documented an average VAS score of 4.9/10. Koo [10] observed a mean VAS of 3.6 with the stamping protocol and 2.7 with the brushing technique. Bonan [13], employing a 5‐point VAS scale, noted a mean score of 4.2 in the stamping mode and 1.5 in the brushing mode. Taken together, these findings underscore the superior comfort and high efficacy of the DMC cooling system compared to previously used modalities.

At the 6‐month follow‐up, effective hair removal was confirmed by three blinded assessors across both treatment protocols. The mean hair reduction score, based on the scoring criteria outlined in Table 3, was 3.02 for areas treated with the Avalanche method and 2.98 for sites where stamping was used during the final three sessions, with no statistically significant difference observed between the sides. These findings align with our previous study, in which only axillary regions were treated and yielded similar outcomes [12]. In comparison, Bonan reported slightly higher hair reduction rates—87% with the stamping technique and 78% with brushing—though these results followed eight treatment sessions [13]. Gold documented approximately 45% hair reduction at 3‐month follow‐up using a multi‐wavelength laser system (755, 810, and 1064 nm) after four sessions of brushing (In‐Motion) [8]. A similar study conducted by Koo involved five treatment sessions, assessing hair reduction outcomes 6 months post‐treatment. The results revealed average hair reduction rates of 33.5% for the stamping method and 40.7% for treatments performed using a brushing motion, relative to baseline values [10].

In our study, patients did not express a distinct preference between the stamping and brushing/Avalanche treatment modalities. Other studies we identified that directly compared brushing and stamping techniques were conducted by Bonan [13], Moftah [14] and Bennardo [18]. In all studies, the majority of patients indicated a preference for the brushing mode, with Bonan reporting statistically significant results [13] and Bennardo and Moftah reporting improved comfort [14, 18]. Bonan also reported a satisfaction score of 3.75 on a 1–5 scale, where 3 corresponds to “satisfied” and 4 to “very satisfied,” following four sessions of brushing‐mode hair removal (In‐Motion) at the 3‐month follow‐up [8]. This result is consistent with our PGI‐I score of 1.65 (on a 7‐point scale) at the 6‐month follow‐up, where 1 represents “very much improved” and 2 indicates “much improved.” The absence of a significant difference in patient preference between the two treatment approaches may be attributed to the advanced DMC cooling system employed in this study, which likely enhanced comfort even during stamping sessions. Additionally, stamping was introduced only in the final three treatment sessions, a phase characterized by markedly reduced hair density and thickness, which may have further minimized discomfort typically associated with this protocol.

Only transient erythema—commonly expected following laser hair removal [17]—was observed in our study. No serious or persistent adverse effects were reported, consistent with findings from previous studies [1, 8, 10]. In contrast, Bonan [13] reported first‐degree burns in 9.2% of patients treated with the stamping technique and 1.8% of those treated with the brushing method, though no other major complications were noted. Additionally, Moftah [14] observed cases of hypo‐ and hyperpigmentation, likely influenced by the darker skin tone of her patient cohort.

The limitations of this study include a relatively small sample size, a predominance of female participants, the absence of randomization in the allocation of treated sides, and the lack of objective, quantitative assessments of hair growth and density. These factors may influence the generalizability and methodological robustness of the findings. In addition, the follow‐up period of 6 months represents only a medium‐term assessment; a 12‐month or longer follow‐up is typically required to evaluate long‐term efficacy and to substantiate claims of permanent hair‐reduction outcomes.

## 5 Conclusion

Previous studies have demonstrated that coarse and dense hair absorbs greater amounts of laser energy, which can lead to increased heat generation and heightened patient discomfort during treatment [19, 20]. Based on these findings, we recommend initiating hair removal with the Avalanche brushing protocol in patients exhibiting increased sensitivity and/or thick, dense hair, particularly during the early treatment sessions. As hair density and thickness diminish over the course of therapy, transitioning to the stamping protocol may be appropriate. This study supports the efficacy of such a combined approach, demonstrating that it can streamline treatment duration without compromising patient comfort.

## Funding

There was no external funding for this study. The author, Anže Zorman, is partially employed by Fotona—the manufacturer of the laser device used in this research—in a role limited to education and product demonstrations. This employment had no involvement in, or influence on, the design, conduct, analysis, or reporting of the present study.

## Conflicts of Interest

A.Z. is engaged in part‐time employment with Fotona d.o.o., serving in the roles of researcher, lecturer, and educator.

## Data Availability

The data that support the findings of this study are available on request from the corresponding author. The data are not publicly available due to privacy or ethical restrictions.
